# The Virulence Factor p25 of Beet Necrotic Yellow Vein Virus Interacts With Multiple Aux/IAA Proteins From *Beta vulgaris*: Implications for Rhizomania Development

**DOI:** 10.3389/fmicb.2021.809690

**Published:** 2022-01-24

**Authors:** Maximilian M. Muellender, Eugene I. Savenkov, Michael Reichelt, Mark Varrelmann, Sebastian Liebe

**Affiliations:** ^1^Institute of Sugar Beet Research, Department of Phytopathology, Göttingen, Germany; ^2^Department of Plant Biology, Uppsala BioCenter SLU, Swedish University of Agricultural Sciences, Linnean Center for Plant Biology, Uppsala, Sweden; ^3^Max Planck Institute for Chemical Ecology, Department of Biochemistry, Jena, Germany

**Keywords:** protein-protein interaction, p25, beet necrotic yellow vein virus, auxin, *Beta vulgaris*, plant virology, Aux/IAA

## Abstract

Rhizomania caused by Beet necrotic yellow vein virus (BNYVV) is characterized by excessive lateral root (LR) formation. Auxin-mediated degradation of Aux/IAA transcriptional repressors stimulates gene regulatory networks leading to LR organogenesis and involves several Aux/IAA proteins acting at distinctive stages of LR development. Previously, we showed that BNYVV p25 virulence factor interacts with BvIAA28, a transcriptional repressor acting at early stages of LR initiation. The evidence suggested that p25 inhibits BvIAA28 nuclear localization, thus, de-repressing transcriptional network leading to LR initiation. However, it was not clear whether p25 interacts with other Aux/IAA proteins. Here, by adopting bioinformatics, *in vitro* and *in vivo* protein interaction approaches we show that p25 interacts also with BvIAA2 and BvIAA6. Moreover, we confirmed that the BNYVV infection is, indeed, accompanied by an elevated auxin level in the infected LRs. Nevertheless, expression levels of BvIAA2 and BvIAA6 remained unchanged upon BNYVV infection. Mutational analysis indicated that interaction of p25 with either BvIAA2 or BvIAA6 requires full-length proteins as even single amino acid residue substitutions abolished the interactions. Compared to p25-BvIAA28 interaction that leads to redistribution of BvIAA28 into cytoplasm, both BvIAA2 and BvIAA6 remained confined into the nucleus regardless of the presence of p25 suggesting their stabilization though p25 interaction. Overexpression of p25-interacting partners (BvIAA2, BvIAA6 and BvIAA28) in *Nicotiana benthamiana* induced an auxin-insensitive phenotype characterized by plant dwarfism and dramatically reduced LR development. Thus, our work reveals a distinct class of transcriptional repressors targeted by p25.

## Introduction

Beet necrotic yellow vein virus belongs to the genus *Benyvirus* within the family *Benyviridae* and is the causal agent of rhizomania disease in sugar beet (Tamada and Abe, 1989; Tamada et al., 1989; Gilmer et al., 2017). Rhizomania was first described in Italy in the early 1950s and spread to almost all sugar beet-growing areas worldwide in the past decades (McGrann et al., 2009; Liebe et al., 2016). The virus causes leaf symptoms, such as yellowing and vein necrosis. Most important, however, are the severe symptoms induced in the infected taproots characterized by reduced size and wineglass shape, necrosis of the vascular tissue and massive lateral root (LR) proliferation, termed as root beard (Tamada and Abe, 1989). These root symptoms cause dramatic reduction of taproot weight and massive yield losses, making BNYVV to be one of the most important viral pathogens in sugar beet cultivation. BNYVV is naturally transmitted by the soil-borne plasmodiophoromycete *Polymyxa betae* Keskin which can persist in soil for decades (Tamada and Kondo, 2013). Nowadays, the only efficient way to control rhizomania disease is the cultivation of resistant sugar beet varieties.

Beet necrotic yellow vein virus has a multipartite genome comprising four to five positive-sense, single stranded RNA segments. Each RNA is capped at the 5′ end and polyadenylated at the 3′ end. RNA1 possesses one open reading frame (ORF) encoding an RNA-dependent RNA polymerase with motifs for methyltransferase, helicase and a papain-like protease (Bouzoubaa et al., 1987; Richards and Tamada, 1992; McGrann et al., 2009). RNA2 contains six ORFs, encoding a coat protein (CP), a CP-read-through (CP-RT) protein, a triple gene block (TGB) of movement proteins and a small 14 kDa cysteine-rich protein, a viral suppressor of RNA silencing (Tamada and Kusume, 1991; Chiba et al., 2013). RNA3 encodes the p25 protein, the virulence factor that is required for systemic infection in *Beta* species and symptom development (Tamada et al., 1989; Koenig et al., 1991; Lauber et al., 1998).

The massive proliferation of lateral roots (LR) upon BNYVV infection relies on the presence of p25 (Koenig et al., 1991; Tamada et al., 1999; Peltier et al., 2011). In general, the development of LRs is controlled by the phytohormone auxin and its tightly regulated transport and signaling pathways (Gray et al., 2001; Dharmasiri et al., 2005; Ori, 2019). Aux/IAA proteins are key regulators within this auxin signaling pathway as they inhibit the transcriptional activity of auxin response factors (ARFs) under low auxin concentration (Luo et al., 2018). In turn, ARFs are transcription factors regulating the expression of auxin-responsive genes by binding to auxin-responsive elements (AREs) within the promotors (Chandler, 2016; Li et al., 2016). Aux/IAA proteins are rapidly degraded when the cellular auxin level increases. This leads to a release of ARFs, regulating the expression of auxin-responsive genes (Leyser, 2018). Interestingly, the sugar beet taproot undergoes massive reprogramming of auxin-responsive genes upon BNYVV infection (Schmidlin et al., 2008; Gil et al., 2018, 2020). This includes the LATERAL ORGAN BOUNDARIES DOMAIN (LBD) transcriptional network as well as expression of EXPANSINS (EXPs), all of which are important for LR development (Liu et al., 2005; Lee and Kim, 2013; Lee et al., 2015). In a sugar beet cDNA library screen (Thiel and Varrelmann, 2009), we identified the Aux/IAA protein BvIAA28 (also known as BvAUX28) as a putative interaction partner of p25. Further characterization showed that p25 interacts with BvIAA28 *via* domains I and II (Gil et al., 2018). Additionally, the co-expression of both proteins revealed that p25 inhibits the nuclear localization of BvIAA28. It has been assumed that the p25-mediated translocation of BvIAA28 into the cytoplasm deprives the protein of its repressor activity in the nucleus leading to an up-regulation of auxin-responsive-genes that are under the control of BvIAA28.

The discovery that the p25 virulence factor interacts with a sugar beet Aux/IAA protein (BvIAA28) (Thiel and Varrelmann, 2009; Gil et al., 2018) prompted us to test the other BvAux/IAAs proteins for their potential interaction with p25 employing three independent methods, namely, yeast two-hybrid system (Y2H), bimolecular fluorescence complementation (BiFC) and co-immunoprecipitation (co-IP). This study identified two additional Aux/IAA proteins - BvIAA2 and BvIAA6 - interacting with p25. Further analysis revealed that p25 sequesters negative regulators of LR initiation and development suggesting activation of a transcriptional network leading to LR induction. This study expands the repertoire of the p25-interacting partners and their potential role in development of rhizomania syndrome.

## Materials and Methods

### Beet Necrotic Yellow Vein Virus Sugar Beet Inoculation

The BNYVV susceptible sugar beet genotype KWS03 (KWS Saat SE, Einbeck, Germany) was used for infection with BNYVV. Young sugar beet seedlings were mechanically inoculated with the BNYVV A-type infectious clone (Laufer et al., 2018) according to Liebe et al. (2020). All plants were kept under controlled greenhouse conditions (24°C/14 h light, 18°C/10 h dark). BNYVV infection and measurement of relative virus contraction in lateral roots was determined by means of DAS-ELISA (DSMZ, AS-0737, Brunswick, Germany) as described by Liebe et al. (2020).

### Auxin Quantification

To measure the auxin content in healthy and BNYVV infected sugar beet roots, 250 mg of homogenized root cortex and lateral root tissue per sample was used. Auxin (indole-3-acetic acid) was extracted with 1 ml methanol containing 40 ng of D5-indole-3-acetic-acid (OlChemIm s.r.o, Olomouc, Czechia) at 42 and 66 dpi. The experiment was performed in eight biological replicates. Samples were analyzed using liquid chromatography (Agilent 1260 Infinity Quaternary LC system, Agilent Technologies, Santa Clarita, California) coupled to a triple quadrupole mass spectrometer (LC-MS/MS). Separation was achieved on a Zorbax Eclipse XDB-C18 column (50 mm × 4.6 mm, 1.8 μm, Agilent Technologies). Formic acid (0.05%) in water and acetonitrile were employed as mobile phases A and B, respectively. The elution profile was: 0–0.5 min, 10% B in A; 0.5–4 min, 10–90% B in A; 4.1–4.5 min 100% B and 4.6–7 min 10% B in A. The mobile phase flow rate was 1.1 ml min^–1^. The column temperature was maintained at 25°C. The liquid chromatography was coupled to a QTRAP 6500 tandem mass spectrometer (AB Sciex, Darmstadt, Germany) equipped with a Turbospray ion source operated in positive ionization mode. The ionspray voltage was maintained at 5,500 eV. The turbo gas temperature was set at 650°C. Nebulizing gas was set at 60 psi, curtain gas at 40 psi, heating gas at 60 psi and collision gas at medium. Multiple reaction monitoring was used to monitor analyte parent ion → product ion: m/z 176 → 130 for indol-3-acetic acid; m/z 181 →133 + m/z 181 →134 + m/z 181 →135 for D5-indol-3-acetic acid. Collision energy was 19V; declustering potential was 20V. Both Q1 and Q3 quadrupoles were maintained at unit resolution. Analyst 1.5 software (Applied Biosystems) was used for data acquisition and processing.

### Yeast Two Hybrid

To identify protein-protein interaction of p25 with the Aux/IAA Proteins from *B. vulgaris*, a yeast two hybrid system (YTH) was used (Fields and Song, 1989). After RNA extraction from BNYVV susceptible sugar beet root material (MACHEREY-NAGEL, Dueren, Germany) and subsequent cDNA synthesis (Thermo Fisher Scientific, Waltham, Massachusetts) all sugar beet encoded *Aux/IAA* genes were based on the annotated sequence from the Kyoto Encyclopedia of Genes and Genomes (KEGG) (Supplementary Table 1). The *Aux/IAA* genes were cloned into pJG4-5 vectors with C-terminal B42 transcription activation domain-HA epitope (AD-Aux/IAA) as prey. The viral pathogenicity factor from BNYVV, *p25* was cloned into pEG202 with CDS1 LexA DNA binding domain (BD-p25) as bait. All plasmids were generated using standard restriction enzyme cloning (Thermo Fisher Scientific). After transformation into chemically competent DH5α *E. coli* cells (Inoue et al., 1990), all plasmids were verified by commercial capillary Sanger sequencing (Microsynth Seqlab, Goettingen, Germany). The constructs were super transformed into the high sensitivity strain *S. cerevisiae* EGY48: MATα, *trp1*, *his3*, *ura3*, *leu2*:*6 LexAop*-*LEU2* using a lithium acetate/single-stranded carrier DNA/polyethylene glycol method (Gietz and Woods, 2002). The GFP plasmid pGNG1 was omitted, because no screen was performed to identify unknown interaction partners and it was not necessary to select for green florescent colonies. All recipes were taken from the Origene *DupLEX-A* user’s manual and modified according to the individual requirements. The lacking amino acids in the drop out media were indicated by the single-letter amino acid code. BD-p25 with each AD-Aux/IAA AD-Aux/IAA were co-transformed to test for interaction and BD-p25 or BD-IAA transformed with the AD or BD without any fusion proteins, respectively, served as control for autoactivation. Three colonies were then individually resuspended according to protocol and diluted in water. Then, 5 μl of the dilution series (1 × 10^–1^-1 × 10^–4^) was spotted on DOBA (gal/raf) -H, -W as growth control, DOBA (gal/raf) -H, -W, -L as interaction or as autoactivation medium, respectively. The positive control AD-p53 with BD-LTA and the negative control AD(-empty) with BD(-empty) were supplied by MoBiTech (Göttingen, Germany). The growth controls were incubated at 30°C for about 3–4 days, the interaction- and autoactivation controls for about 5–6 days.

### Preparation of *R. radiobacter* for Agroinoculation

Electrocompetent cells of the *Rhizobium radiobacter* (syn. *Agrobacterium tumefaciens*/*Agrobacterium fabrum*) strain C58/C1 were used for transformation of all plasmids, used in this work (Voinnet et al., 1998).

### Bimolecular Fluorescence Complementation Assay

To verify the results from YTH, bimolecular fluorescence complementation assay (BiFC) was used according to Jach et al. (2006), Zilian and Maiss (2011). The Aux/IAA candidates were fused C- and N-terminally to the N-terminal part of mRFP (mRFPN) and p25 was fused in both orientations to mRFPC by one-step cloning isothermal Gibson assembly (Gibson et al., 2009). The constructs were inoculated with an OD_600_ of 0.7 into leaves of four- to 5-week-old *N. benthamiana* wild type plants. Fluorescence in the leaf patches was assessed microscopically at 4 dpi by epifluorescence microscopy at 4 dpi. Positive and negative controls were taken from the BiFC assay (Zilian and Maiss, 2011).

### Co-immunoprecipitation

For final confirmation of the protein interaction results, *in planta* co-immunoprecipitation (co-IP) was chosen. Both Aux/IAA proteins IAA2 and IAA6 and p25 were cloned into the plant expression vector pDIVA (Acc. No. KX665539) under control of CaMV 35S promotor. Additionally, mutants encoding degradation resistant protein variants (BvIAA2_*P*162*L*_, BvIAA6_P64*L*_) allowing higher protein accumulation were created by PCR mutagenesis and subsequent sequencing (Worley et al., 2000). These protein variants were generating using PCR mutagenesis and confirmed by sequencing. To further increase the expression of the Aux/IAAs, a Tobacco etch virus (TEV) translational enhancer sequence (Zilian and Maiss, 2011) was inserted upstream of the *Aux/IAA* genes. The Aux/IAA proteins were fused to the N-terminus of a 3xFLAG tag (DYKDDDDK) and a single HA tag (YPYDVPDYA) was fused to the p25 C-terminus. Three days after infiltration of *N. benthamiana* leaves with the constructs, the patches were harvested and grounded in liquid nitrogen to a fine powder. The powder was mixed 1:1 (w/v) with extraction buffer (Sacco et al., 2007; Sohn et al., 2014) supplied with 50 μM Mg132 (Sigma-Aldrich, St. Louis, Missouri) to prevent proteasome mediated protein degradation. After incubation on ice for 5 min, the reaction tubes were centrifuged (5,000 *g*) for 15 min at 4°C and the supernatant was used as input. For immunoprecipitation, 500 μl of the input was mixed with 25 μl equilibrated Pierce™ Anti-HA Magnetic Beads (Thermo Fisher Scientific, Waltham, Massachusetts) and incubated for 1 h at 4°C. After three washing steps, all bound proteins to the beads were eluted using 2x Laemmli buffer (Bio-Rad Laboratories, Hercules, CA, United States) and incubated for 5 min at 95°C. Additionally, the proteins were detected in the input. All samples were checked using SDS polyacrylamide gel electrophoresis and immunoblotting.

### Subcellular Localization

To determine the subcellular localization of BvIAA2 and BvIAA6, both genes were fused by Gibson assembly to GFP containing an HA tag. To investigate the effect of p25 on the subcellular localization of Aux/IAAs, only an HA tag was added to minimize negative effects of long attachments. Additionally SV40 NLS was fused to dsRed and served as plant nuclear marker (Kalderon et al., 1984; Lassner et al., 1991). The plasmid pDIVA was used as backbone for the cloning of the localization plasmids (Laufer et al., 2018). Co-expression was performed by means of agroinfiltration with an OD_600_ of 0.7 into *N. benthamiana* leaves. Fluorescence in the leaf patches was assessed microscopically at 4 dpi. The HA tag used to verify protein expression *via* immunodetection.

### Domain Mapping of p25 and Interacting Aux/IAA Proteins

To identify interacting domains of p25 A-type with Aux/IAA proteins, five amino acids were randomly inserted over the whole protein using the Mutation Generation System Kit (F701—Thermo Fisher Scientific). To check for interacting domains of the Aux/IAA proteins, the previously described domains were used (DI-DIV). BvIAA2 and BvIAA6 were separated into two parts (DI-II and DIII-IV) and the described domains were deleted individually (DII-IV; DI, III, IV; DI, II, IV; DI-III). For IAA2 primers were designed to delete the domains between amino acid positions AA 102/103, AA 189/190 and AA 252/253 and the primers for IAA6 were designed to delete the domains between amino acids AA 41/42, AA 76/77 and AA 132/133. The deletions were introduced into the respective YTH and BiFC plasmids by PCR mutagenesis with subsequent sequencing.

### Confocal Laser Scanning Microscopy

To visualize protein fluorescence, confocal laser scanning microscopy (CLSM) was used. The mRFP and GFP fluorescence was visualized with the TCS-SP5 confocal laser-scanning microscope (Leica Microsystems). Excitation/emission wavelengths for mRFP were 566 nm/515–523 nm and for GFP the wavelengths were 488 nm/515–523 nm. All confocal images were processed with the LAS-AF software version 2.6.3.8173 (Leica Microsystems, Wetzlar, Germany).

### Protein Extraction From Yeast and Plant Tissue, Sodium Dodecyl Sulfate Polyacrylamide Gel Electrophoresis, and Immunodetection

Protein extraction from yeast was carried out as described by and protein extraction of total plant proteins was carried after (Thiel and Varrelmann, 2009). All protein samples were separated by 12% SDS polyacrylamide gel electrophoresis and electroblotted on polyvinylidene diflouride membranes (Roche, Basel, Switzerland) using semi-dry blotting system (Bio-Rad Laboratories). Immunodetection of HA was carried out using anti-HA high-affinity rat monoclonal antibody (Merck KGaA, Burlington, Vermont—11 867 423 001, 1:1,000) and alkaline phosphatase (AP) conjugated goat anti-rat immunoglobulin G (IgG) (whole molecule) (Merck KGaA—A8438, 1:10,000). FLAG (Merck KGaA—F7425, 1:1,000) and LexA (Merck KGaA—06-719, 1:2,500) were detected with polyclonal rabbit antibodies and AP-conjugated goat anti-rabbit polyclonal antibodies (Merck KGaA—A3687, 1/10,000). C-myc (EQKLISEEDL) was probed with anti-C-myc mouse monoclonal IgG (Thermo Fisher Scientific—13-2,500, 1:500) and detected with AP-conjugated goat anti-mouse IgG (Jackson ImmunoResearch Laboratories, West Grove, Pennsylvania—115-055-003, 1:10,000). Signal detection was performed using NBT/BCIP (chromogenic substrates nitroblue tetrazolium chloride and 5-bromo-4-chloro-3′-indolyphosphate) ready-to-use tablets (Merck KGaA).

### Heterologous Expression of Aux/IAA Proteins

For heterologous expression of Aux/IAA proteins, the genes from BvIAA2, BvIAA6 and BvIAA28 were cloned into the infectious TRV RNA2 cDNA clone by Gibson assembly under the control of a subgenomic promoter of the Pea early-browning virus downstream of the TRV-CP (Liu et al., 2002; Ghazala and Varrelmann, 2007; Lindbo, 2007). Additionally, degradation resistant protein variants (BvIAA2_*P*162*L*_, BvIAA6_P64*L*_, BvIAA28_P146*L*_), allowing higher protein accumulation, were generated by PCR mutagenesis (Worley et al., 2000). A TRV RNA2 expressing mRFP (RNA2-mRFP) was used as control to distinguish symptoms of candidate gene overexpression from general TRV symptoms and to check for systemic infection. For systemic TRV infection, the leaves of 14-days-old *N. benthamiana* seedlings were inoculated with an OD_600_ of 0.5 of RNA1 and each RNA2 construct. The root and leaf phenotypes were examined at 33 days postinoculation (dpi). A systemic TRV infection was confirmed by RNA extraction, cDNA synthesis, and final PCR of heterologous expressed Aux/IAA proteins in *N. benthamiana* leaves.

### RT-qPCR Analysis

*BvIAA2*, *BvIAA28* and *BvIAA6* expression was relatively quantified in cDNA of the LR tissue of sugar beet using reverse transcriptase quantitative PCR (RT-qPCR). After RNA extraction (MACHEREY-NAGEL) and cDNA synthesis (Thermo Fisher Scientific) samples were analyzed using iTaq™ Universal SYBR^®^ Green Supermix (#1725121—Bio-Rad Laboratories). Oligonucleotides were designed with NCBI primer-BLAST (listed in Supplementary Table 2). The expression of both *Aux/IAA* genes was quantified relative to the housekeeping genes *glyceraldehyde 3-phosphate dehydrogenase* (*GAPDH*, XM_010679634.2) and *elongation factor 1*β (*EEF1B2*, NM_001303081.2). All qPCR reactions were performed with a C1000 Touch™ Thermal cycler equipped with a CFX96™ Real Time System (Bio-Rad Laboratories). RT-qPCR conditions were as follows: an initial denaturation of 95°C for 3 min followed by 40 cycles of 95°C for 30 s, 60°C for 20 s, 72°C for 30 s, final extension of 72°C for 5 min. All three biological replicates were analyzed in two technical replicates. The Ct values and curves for analysis were generated by the CFX Manager™ Software (Bio-Rad Laboratories) and data normalization and calculation of relative expression values was done using the 2^–Δ*Ct*^ method (Livak and Schmittgen, 2001). The statistical independence between root tissue and leaf tissue was calculated for the individual ΔCt values at each time point.

### Bioinformatic Analysis

Multiple protein sequences alignments, as well as maximum likelihood trees were generated using Geneious 2020.1 software (default settings—Biomatters). Protein sequences of the *B. vulgaris* (BvIAA) and *A. thaliana* (AtIAA) Aux/IAA proteins were downloaded from KEGG database and alignments of sequences were generated using the ClustalW algorithm (default settings—Biomatters).

### Statistical Analysis

Statistical analysis was performed with SigmaPlot14 (SigmaPlot 14.0, Systat Software Inc.). The data were first tested for normal distribution (*p* ≤ 0.05) using Kolmogorov-Smirnov test followed by Brown-Forsythe test to check for equality of group variances (*p* > 0.05). The data were analyzed using Student’s *t*-test. When equality of variances cannot be assumed, Welch’s *t*-test was used. Graphic representations of the data were created using Excel 2013 (Microsoft Corp.). In each graph, the standard deviation (SD) and significance (not significant (n.s.) = *p* > 0.05; *0.01 ≤ *p* < 0.05; ^**^0.001 ≤ *p* < 0.01; ^***^p < 0.001) are displayed. Significant differences between several variants on one factor were performed using one-way ANOVA. Data in tables are presented as mean values ± SD (standard deviation).

## Results

### Phylogenetic and Functional Analysis of the Aux/IAA Proteins From *B. vulgaris*

**FIGURE 1:**
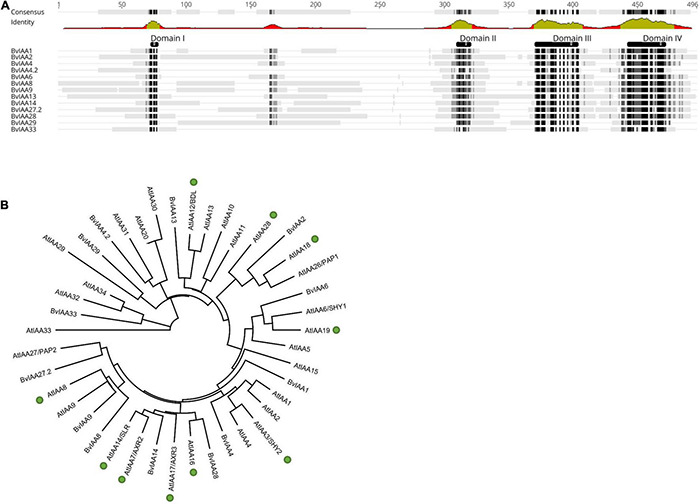
**(A)** Multiple sequence alignment (Geneious 2020.1 software) of all IAA proteins from sugar beet (BvIAA). Black and gray shades in the sequences and green regions of graph below the consensus indicate a high homology of the protein sequences. The functional domains of the proteins are also shown above the sequences (DI-DIV). **(B)** Maximum likelihood tree of all BvIAA proteins and all Aux/IAA proteins from *A. thaliana* (AtIAA). Proteins involved in root formation in *A. thaliana* are highlighted with green circles (after Reed, 2001; Luo et al., 2018).

The Aux/IAA protein BvIAA28 interaction with p25 was previously identified from a screening using a sugar beet cDNA library prepared from a resistant genotype that prevents efficient virus replication and massive lateral root proliferation upon BNYVV infection (Thiel and Varrelmann, 2009). Furthermore, the massive transcriptional reprogramming of auxin-responsive genes observed in a susceptible genotype (Gil et al., 2020) prompted us to hypothesize that p25 might interact with numerous Aux/IAAs. Therefore, to address this hypothesis, we set up a screen with all known Aux/IAAs from sugar beet. Using the KEGG GENOME database, 12 potential Aux/IAA candidates were identified (Supplementary Table 1). Transcripts of the candidate genes from a BNYVV susceptible genotype were sequenced and a multiple sequence alignment and phylogenetic analysis performed. The alignment clearly showed the presence of all four canonical Aux/IAA domains in all candidates except for two. BvIAA4.2 and BvIAA33 appeared not to contain domain II and, thus, were assigned to the class of non-canonical Aux/IAA proteins (Figure 1A). A maximum likelihood tree of the Aux/IAA candidates from *B. vulgaris* together with all known 29 Aux/IAA proteins from *Arabidopsis thaliana* was computed to define potential clades and orthologous groups based on the similarity to corresponding Arabidopsis proteins (Liscum and Reed, 2002; Overvoorde et al., 2005; Luo et al., 2018). Most BvIAA proteins clustered together with the corresponding *Arabidopsis* proteins into ten clades of putative functional homologs (Figure 1B). Notably, BvIAA2, BvIAA4, BvIAA6, BvIAA8, BvIAA9, BvIAA13, BvIAA14 and BvIAA28 proteins clustered together with *Arabidopsis* Aux/IAA proteins involved in root development (Figure 1B, green circles), only BvIAA29 and BvIAA33 fell into other clades (Reed, 2001; Luo et al., 2018). Nevertheless, we tested all Aux/IAA proteins from sugar beet for interaction with p25.

### Interaction Studies of p25 With the Aux/IAA Proteins From *B. vulgaris*

**FIGURE 2:**
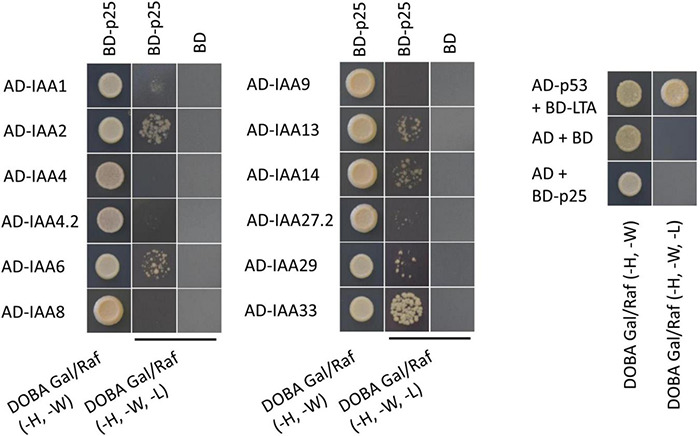
Results from YTH experiment with all Aux/IAA proteins from sugar beet and BNYVV p25. The positive control AD-p53 with BD-LTA and the negative control AD(-empty) with BD(-empty) were supplied by MoBiTech. BNYVV p25 was fused to the BD and the IAAs to the AD to test for interaction. Yeast transformants, containing both plasmids were selected on DOBA Glu (-H, -W), single colonies were resuspended in water and diluted 1 × 10^–1^- 1 × 10^–4^. 5 μl of each dilution was spotted on the control medium [DOBA Glu (-H, -W)] and selection medium [DOBA Gal/Raf (-H, -W, -L)], only the 1 × 10^–2^ dilution is shown here. An AD or BD without any fusion proteins and transformed with BD-p25 or AD-Aux/IAA, respectively, served as control for autoactivation. AD, activating domain; BD, binding domain; DOBA, Dropout Base Agar.

**FIGURE 3:**
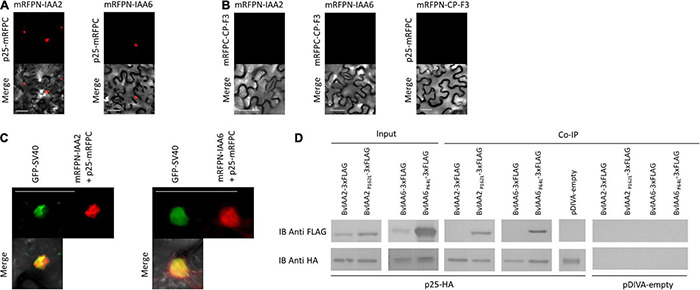
Confirmation of the BNYVV p25 interaction with IAA2 and IAA6 by bimolecular fluorescence complementation and co-immunoprecipitation. The candidates were co-expressed in *N. benthamiana* leaves by *A. tumefaciens* C58C1 cells harboring **(A)** pCB:p25-mRFPC/pBiFC-mRFPN-IAA2 or pCB:p25-mRFPC/pCB:mRFPN-IAA6 to test the interaction and **(B)** pCB:mRFPC-CP-F3/pCB:mRFPN-IAA2, pCB:mRFPC-CP-F3/pCB:mRFPN-IAA2 or pCB:p25-mRFPC/pCB:mRFPN-CP-F3 to test for autoactivation of the fusion proteins. **(C)** Additionally, the interacting BiFC partners were co-expressed with the nuclear marker pDIVA:GFP-SV40 to confirm the nuclear localization of the interaction. Images were taken at 4 dpi. Scale bars, 50 μm. **(D)** Immunoblot (IB) showing the Aux/IAA proteins coimmunoprecipitated with p25. The total proteins were isolated from *Agrobacterium*-infiltrated *N. benthamiana* leaves expressing the Aux/IAAs-3xFLAG (up) and p25-HA (low). The input is shown at the left and the and immunoprecipitated samples with anti-HA antibodies are shown in the right. The candidates, co-infiltrated with pDIVA-empty were used as controls, to detect unspecific binding.

To determine whether the Aux/IAA from sugar beet interact with p25, a Y2H experiment was performed. The analysis revealed that BvIAA2, BvIAA6, BvIAA13, BvIAA14, BvIAA29 and BvIAA33 could potentially interact with p25 and none of these proteins displayed autoactivation of Y2H-inducible reporter (Figure 2). The six interactors were selected for further validation using bimolecular fluorescence complementation (BiFC) (Zilian and Maiss, 2011) in *N. benthamiana* leaf tissue. The BiFC experiments showed that among the six candidates tested, only BvIAA2 and BvIAA6 interact with p25 *in planta*. Moreover, these interactions could only be detected when p25 was fused C-terminally to mRFP-C and the Aux/IAA candidates were fused N-terminally to mRFP-N (Figure 3A). Again, no autoactivation of BvIAA2, BvIAA6 or BvIAA28 was detected (Figure 3B). Co-expression of the abovementioned BiFC constructs with the nuclear marker GFP-SV40 revealed that the interactions of p25 with both Aux/IAAs are strongly restricted to the nucleus (Figure 3C). The interactions of p25 with BvIAA2 and BvIAA6 were also confirmed by co-IP experiments in *N. benthamiana* leaves (Figure 3D). However, only the interaction of p25 with the degradation-resistant variants of the Aux/IAA proteins could be detected (Figure 3D). Notably, in the input samples, the accumulation of the unmodified wt Aux/IAA proteins was much low compared to the degradation-resistant variants suggesting fast turnover of the BvIAA2 and BvIAA6 proteins as expected. Finally, the expression of all BvIAA proteins tested and p25 in all three assays was confirmed by immunoblotting (Supplementary Figures 1, 2).

### Mapping of Interacting Sites in p25 and Aux/IAAs

To identify amino acid residues in BvIAA6, BvIAA2 and p25 involved in the protein interactions fourteen *p25* mutants were generated by pentapeptide scanning mutagenesis. The expression of these mutants results in a single five amino acid insertion randomly distributed along the sequence of the protein (Table 1). Seven randomly chosen p25 mutants were tested for interaction with BvIAA2 and nine randomly chosen mutants were assessed for interaction with IAA6 in Y2H system. Two of the mutants were tested with both BvAux/IAA proteins to confirm the validity of the results for both candidates. Surprisingly, these experiments showed that none of the p25 mutants interacted (Table 1) neither with BvIAA2 nor with BvIAA6. To further verify the interaction of p25 with BvIAA2 and BvIAA6, three p25 mutants of those tested above were used in BiFC experiments. The results confirmed the Y2H experiments, no interaction of the p25 mutants with BvIAA2 or BvIAA6 could be detected (data not shown). As before, the protein expression of wild type p25 and the expression of four randomly selected p25 mutants was confirmed by immunoblotting (Supplementary Figure 3).

**TABLE 1:** Results of the YTH assay of the p25 pentapeptide scanning mutants tested for interaction with IAA2 and IAA6.

p25 variety	IAA2	IAA6
p25 wt	✓	✓
p25.Val51_Tyr52ins5	X	n.d.
p25.Gly119_Leu120ins5	X	n.d.
p25.Val130_Pro131ins5	X	n.d.
p25.Val140_Asp141ins5	X	n.d.
p25.Val178_Asn179ins5	X	n.d.
p25.Val81_Met82ins5	X	X
p25.Asp200_Val201ins5	X	X
p25.Cys31_Arg32ins5	n.d.	X
p25.Arg62_Gly63ins5	n.d.	X
p25.Pro93_Ile94ins5	n.d.	X
p25.Asn118_Gly119ins5	n.d.	X
p25.Val121_Ile122ins5	n.d.	X
p25.Leu132_His133ins5	n.d.	X
p25.Asn156_Ala157ins5	n.d.	X

*Checkmark (✓) = positive interaction, cross (X) = no interaction, n.d. = not determined.*

To reveal which domains of BvIAA2 and BvIAA6 are required for the interaction with p25, six constructs for each BvIAA protein expressing various sets of the conserved domains I to IV were tested by Y2H assays and BiFC (Table 2). In both experiments, no interaction was detected with either the Y2H test or BiFC (Table 2), showing that deletion of any domain of BvIAA2 or BvIAA6 results in loss of interaction with p25. Similar to the p25 mutants as described above, it was found that deletion of any domain of BvIAA2 or BvIAA6 resulted in loss of interaction with p25. Only wt IAA2 and IAA6 showed stable interaction with p25 in YTH and BiFC (Table 2).

**TABLE 2:** Results of the YTH and BiFC assays with the different domain variants of IAA2 and IAA6 with p25 wt.

IAA	YTH	BiFC
IAA2	✓	✓
IAA2 DI + II	X	X
IAA2 DIII + IV	X	X
IAA2 DII, III, IV	X	X
IAA2 DI, III, IV	X	X
IAA2 DI, II, IV	X	X
IAA2 DI, II, III	X	X
IAA6	✓	✓
IAA6 DI + II	X	X
IAA6 DIII + IV	X	X
IAA6 DII, III, IV	X	X
IAA6 DI, III, IV	X	X
IAA6 DI, II, IV	X	X
IAA6 DI, II, III	X	X

*Checkmark (✓) = positive interaction, cross (X) = no interaction.*

We then investigated whether alterations (amino acid residue substitutions) in the nuclear localization signal (NLS) and nuclear export signal (NES) (Vetter et al., 2004) of p25 affect the interaction with BvIAA2 and BvIAA6. To this end, the p25 NLS motif ^57^KRIRFR^62^ was replaced with either ^57^AAIAFA^62^ or ^57^KRIRFA^62^ and the NES motif ^169^VYMVCLVNTV^178^ was altered to ^169^AYMACLVNTV^178^ (Vetter et al., 2004). The Y2H and BiFC experiments showed that interactions with both BvIAA2 and BvIAA6 were lost when either the NLS or NES signal was disrupted (Supplementary Figure 4).

### Subcellular Localization of IAA2 and IAA6 Upon Co-expression With p25

**FIGURE 4:**
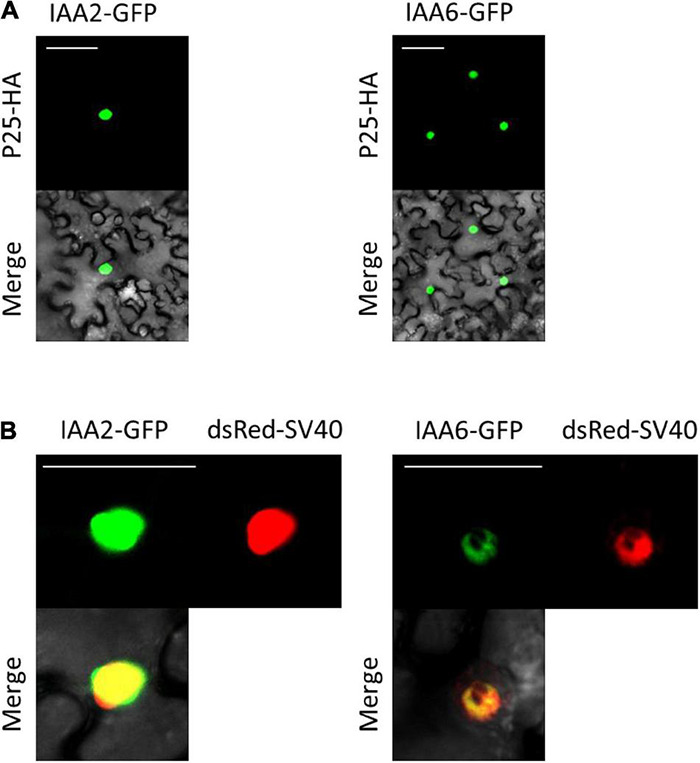
Subcellular localization of interacting Aux/IAAs co-expressed with and without p25 **(A)** Co-infiltration of interacting partners p25 fused to an HA tag (p25-HA) together with IAA2 fused to GFP (IAA2-GFP) or IAA6 fused to GFP (IAA6-GFP). **(B)** Subcellular localization of IAA2-GFP and IAA6-GFP transiently expressed in *N. benthamiana* epidermal leaf cells. Both proteins were co-expressed with the nuclear marker dsRed-SV40. Images were taken at 4 dpi. Scale bars, 50 μm.

Since a previous study reported that the interaction of p25 with BvIAA28 results in p25-mediated translocation of BvIAA28 from the nucleus into the cytoplasm (Gil et al., 2018), we investigated whether p25 affects the nuclear accumulation of BvIAA2 and BvIAA6. To minimize protein modifications which can disturb the interaction, the BvIAA2 and BvIAA6 proteins were fused to GFP-HA tag and p25 was fused to a single HA-tag only. Anti-HA antibodies were used to detect the proteins by immunoblotting (Supplementary Figure 5) and the GFP reporter was employed to determine the subcellular localization of the BvIAA2 and BvIAA6 proteins (Figure 4). Additionally, both BvIAA2 and BvIAA6 proteins were co-expressed with dsRed-SV40 to verify their nuclear localization. The localization experiments showed that both, BvIAA2 and BvIAA6 localize to the nucleus regardless of whether they are transiently expressed on their own or co-expressed with p25. To examine an effect of the interaction on p25, the p25 protein was tagged with GFP and co-expressed with either BvIAA2-mRFP or BvIAA6-mRFP. There was no change in the subcellular localization of p25 in the presence of the Aux/IAA proteins, p25 still localized to the nucleus and the cytoplasm (Vetter et al., 2004) (Supplementary Figure 6).

### Measurement of the Indole-3-Acetic Acid Content in Beet Necrotic Yellow Vein Virus Infected Sugar Beet Plants

**FIGURE 5:**
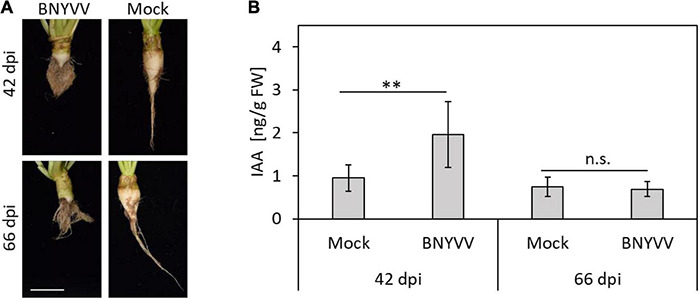
Determination of the IAA content in BNYVV infected sugar beet roots at 42 and 66 dpi by LC-MS/MS. **(A)** Root phenotype of BNYVV mechanically infected vs. non-inoculated (mock) sugar beets after both harvest dates. Scale bar, 5 cm. **(B)** IAA content in the lateral roots and root cortex of BNYVV infected and non-inoculated sugar beet plants. Horizontal bars indicate significance (n.s. = not significant = *p* > 0.05; ** = 0.001 = *p* < 0.01) and vertical bars indicate standard deviation (*n* = 8).

Since BNYVV is thought to interfere with important regulatory nodes of the auxin signaling pathway, changes of the auxin concentrations during BNYVV infection process are expected. To address this question, the auxin (indole-3-acetic acid, IAA) content was measured in the root cortex and lateral roots of healthy and BNYVV-inoculated sugar beet plants 42 and 66 dpi using LC-MS/MS. Accumulation of BNYVV in the inoculated plants was confirmed by ELISA (data not shown) prior to measurements and eight biological replicates (individual plants) of each treatment were selected for auxin quantification. The LC-MS/MS measurements revealed that the auxin content in BNYVV infected roots (1.96 ± 0.76 μg g FW^–1^) was approximately as twice as high compared to healthy sugar beet roots (0.95 ± 0.31 μg g FW^–1^) at 42 dpi (Student’s two-tailed *t*-test, *p* = 0.007). In contrast, the auxin content measured in healthy (0.75 ± 0.23 μg g FW^–1^) and infected (0.69 ± 0.17 μg g FW^–1^) roots at 66 dpi was similar (Student’s two-tailed *t*-test, *p* > 0.05) (Figure 5).

### Quantification of IAA2 and IAA6 Expression in Beet Necrotic Yellow Vein Virus Infected Sugar Beet Plants

**FIGURE 6:**
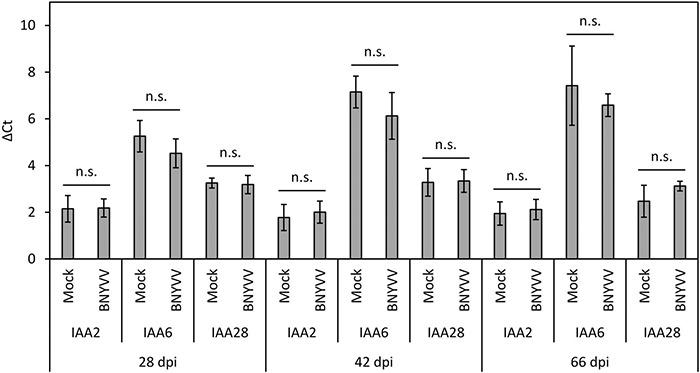
Expression level of *BvIAA2*, *BvIAA6* and *BvIAA28* in BNYVV mechanically infected sugar beet roots compared to the expression in non-inoculated (mock) sugar beet roots. The roots were analyzed at 28, 42, and 66 dpi. Horizontal bars indicate significance (n.s. = not significant) and vertical bars indicate standard deviation (*n* = 4).

Having determined that auxin levels are significantly increased in the BNYVV-infected LR, we next asked whether this dramatic change results in altered expression of *BvIAA2* and *BvIAA6.* To address this question, RT-qPCR was conducted for *BvIAA2*, *BvIAA6* and *BvIAA28* using total RNAs of mock-inoculated and virus-infected sugar beet roots at 28, 42 and 66 dpi. But before setting up RT-qPCR experiments, the accumulation of BNYVV in LR of the sugar beet plants selected for RT-qPCR analysis was confirmed by ELISA (Supplementary Figure 7). There was no change detected in the expression of either *BvIAA2* or *BvIAA6* at any time point tested (Student’s two-tailed *t*-test, *p* > 0.05; Figure 6). Thus, we concluded that the expression of *BvIAA2*, *BvIAA6* and *BvIAA28* was not affected by BNYVV infection.

### Effect of BvIAA2, BvIAA6 and BvIAA28 Expression on Lateral Root Formation in *N. benthamiana*

**FIGURE 7:**
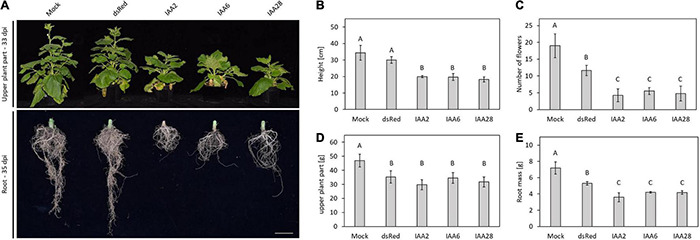
Heterologous expression of Aux/IAAs in *N. benthamiana*. **(A)** Upper plant part and root phenotypes of *N. benthamiana*, non-inoculated (mock), mechanically infected with TRV expressing dsRed, IAA2, IAA6, and IAA28. Pictures of the upper plant part were taken at 33 dpi and pictures of the root phenotype were taken at 35 dpi. Scale bar, 5 cm. The examinations of different plant parts are shown on the right. **(B)** Plant height in cm, **(C)** number of flowers, **(D)** mass of the upper plant part in g, **(E)** mass of the root in g. Data and error bars represent the mean and the standard deviation of at least four replicates (*n* = 4). Significant differences are indicated as letters above the bars.

To elucidate a possible effect of the p25-interacting Aux/IAA proteins (BvIAA2, BvIAA6 and BvIAA28) on LR development we overexpressed *BvIAA2*, *BvIAA6* and *BvIAA28* and characterized the *Aux/IAA*-overexpression phenotypes. Initially, we also planned to perform Aux/IAA-knock down experiments in sugar beet using Tobacco rattle virus (TRV)-based virus-induced gene silencing (VIGS) system. Unfortunately, TRV RNA2 failed to accumulate in sugar beet inoculated roots and only TRV RNA1 was detectable (data not shown) making VIGS or overexpression from a viral vector not possible in sugar beet. Since *N. benthamiana* represents a more genetically tractable model than sugar beet, and both react to changes in auxin-signaling, all subsequent experiments were performed in *N. benthamiana*. Thus, *BvIAA2*, *BvIAA6* and *BvIAA28* were expressed from TRV vector in *N. benthamiana*. Additionally, the degron motif in domain II of BvIAA2, BvIAA6 and BvIAA28 was altered by site-directed mutagenesis to reduce the auxin mediated degradation of the corresponding proteins and to enhance the phenotypic effect of the overexpression (Worley et al., 2000). The obtained *Aux/IAA* mutants were expressed from TRV vector as well. As expected, heterologous expression of BvIAA2, BvIAA6 and BvIAA28 in *N. benthamiana* resulted in phenotypes that resemble auxin-insensitivity characterized by overall dwarfism of the plant (Park et al., 2002; Figure 7A). The plant height, number of flowers and root mass was significantly reduced (Student’s two-tailed *t*-test, *p* < 0.05; Figure 7A) as compared to the TRV-mRFP-infected controls. The plants did not differ significantly (Student’s two-tailed *t*-test, *p* > 0.05; Figure 7A) in any of the traits examined (Figures 7B–E), when phenotypes were compared between various constructs (TRV-*BvIAA2* versus TRV-*BvIAA6* versus TRV-*BvIAA28*). Systemic infections of the plants with the corresponding TRV constructs and stability of the insertions were confirmed by PCR (Supplementary Figure 8B) and sequencing (data not shown).

Expression of the degradation-resistant variants of the Aux/IAA proteins, namely, BvIAA2_*P*162*L*_ and BvIAA8_P146*L*_ resulted in death of the plants (Supplementary Figure 8A). However, plants infected with TRV-*BvIAA6_P64*L*_* survived and were characterized by more severe phenotype compared to those induced by TRV-*BvIAA6* (Supplementary Figure 8A). Hence, the auxin-insensitivity phenotype already observed with the unmodified BvIAA6 could be further enhanced.

## Discussion

The excessive formation of LRs is the characteristic symptom of the rhizomania disease in sugar beet. Since LR formation is controlled by auxin (Fukaki et al., 2007; Lavenus et al., 2013; Du and Scheres, 2018), it seems reasonable to assume that BNYVV interferes with the auxin signaling pathway for which experimental evidence was provided in the previous studies (Larson et al., 2008; Gil et al., 2018). In this study, we confirmed that the infection of sugar beets with BNYVV is accompanied by an increase of the auxin concentration in LRs (42 dpi) (Pollini et al., 1990). Interestingly, such an effect was also observed in transgenic *A. thaliana* plants constitutively expressing p25 (Peltier et al., 2011). Transcriptome analyses also revealed that the genes encoding proteins involved in auxin biosynthesis - such as tyrosine decarboxylase 1, tryptophan aminotransferase-related protein 1 and several *YUCCA* genes - are upregulated in BNYVV- infected plants (Gil et al., 2020). The increased auxin content was not detected at a later stage of infection (66 dpi), which might be explained by plant compensatory mechanisms supporting auxin homeostasis, which is crucial for plant development. Whether this is a reaction of the plant, or if it is a mechanism employed by the virus to support LR formation remains unknown. However, our experiments provide a direct correlation between altered auxin content and the presence BNYVV.

The interaction of the transcriptional repressor BvIAA28 with p25 has already been described and characterized (Thiel et al., 2012; Gil et al., 2018). In this study, two additional p25-interacting partners were identified, namely, BvIAA2 and BvIAA6. The interaction was confirmed in Y2H, BiFC and co-IP experiments. However, the fact that only the interaction of the degradation-stable variants of the Aux/IAA proteins can be detected in co-IP experiments shows how labile the interactions are. In general, Aux/IAAs are very short-lived proteins as long as no alterations done to the protein structure to prevent their degradation (Reed, 2001). All p25-interacting Aux/IAA proteins show significant similarity to their corresponding orthologs from *A. thaliana*. BvIAA2, BvIAA6 and BvIAA28 cluster together with Arabidopsis Aux/IAA proteins involved in LR development and root hair formation (Reed, 2001; Luo et al., 2018). Interestingly, the *Arabidopsis* proteins AtIAA18 and AtIAA28, which cluster together with BvIAA2, and AtIAA1 that clusters together with BvIAA6, are negative regulators (transcriptional repressors) of lateral root formation and their auxin-mediated degradation is required for proper LR development. Expression of degradation stable variants of these proteins reduced lateral root development in *N. benthamiana* even in the presence of exogenously supplemented auxin (Fukaki et al., 2002; Uehara et al., 2008; Notaguchi et al., 2012). Such negative regulators are also found among *Arabidopsis* proteins that cluster together with BvIAA28, namely AtIAA14/SLR and AtIAA16 (Fukaki et al., 2002; Rinaldi et al., 2012). However, expression of degradation stable variants of two other Aux/IAA proteins (AtIAA7/AXR2; AtIAA17/AXR3) from this cluster led to an increased number of lateral roots, indicating an enhanced auxin response (Leyser et al., 1996; Nagpal et al., 2000).

Heterologous expression of sugar beet Aux/IAAs using a TRV vector was employed to characterize the effect *BvIAA2*, *BvIAA6* and *BvIAA28* overexpression in *N. benthamiana*. Unfortunately, similar experiments as well as VIGS could not be performed in sugar beet because of instability of the TRV vector in sugar beet roots (see section “RESULTS”). Expression of either *BvIAA2*, *BvIAA6* or *BvIAA28* from TRV in *N. benthamiana* resulted in very similar phenotypes characterized by dramatic inhibition of root development confirming that the auxin-mediated regulatory pathways are highly conserved across different plant species (sugar beet versus *N. benthamiana*). Additional phenotypes associated with *BvIAA2*, *BvIAA6* or *BvIAA28* expression included a stunting and dwarfism, a significant reduction in the number of flowers, and a reduction of the root mass, as well as an overall root shortening. These effects on plant development and growth were further enhanced when a variant of BvIAA6 resistant to auxin-mediated degradation was expressed. Thus, the phenotypes closely resembled those induced by degradation-stable variants of the corresponding *Arabidopsis* homologs of sugar beet Aux/IAAs described above, i.e., degradation stable variants of AtIAA14/SLR, AtIAA16, AtIAA18, AtIAA19 and AtIAA28 also induced a shortening of the root accompanied by reduction in the number of lateral roots (Fukaki et al., 2002; Uehara et al., 2008; Notaguchi et al., 2012; Rinaldi et al., 2012). The expression of degradation stable AtIAA18 also caused a shortening of the internodes (Fukaki et al., 2002), the phenotype that was also observed in this study, when *BvIAA2*, *BvIAA6* or *BvIAA28* were expressed from TRV vector in *N. benthamiana*. Hence, our findings that all p25 interacting Aux/IAA proteins identified so far affect root development in *N*. *benthamiana* is in agreement with the previous studies in *A. thaliana* showing that several Aux/IAAs are involved in controlling various distinct steps of root development and LR formation (Fukaki et al., 2002; Knox et al., 2003; Lavenus et al., 2013). It is also very likely that these steps of LR development in sugar beet are controlled by functional homologs of corresponding *Arabidopsis* Aux/IAA proteins, yet direct evidence is lacking.

Analysis of BvIAA2, BvIAA6 and BvIAA28 sequences revealed the presence of NLS signals similar to those of other Aux/IAA proteins (Abel et al., 1994; Reed, 2001; Wu et al., 2012; Luo et al., 2018). Indeed, the subcellular localization of BvIAA2 and BvIAA6 revealed that they exclusively accumulate in the nucleus like BvIAA28 (Gil et al., 2018). Moreover, the subcellular localization of BvIAA2 and BvIAA6 proteins co-expressed with p25 did not change and both proteins remained confined to the nucleus in the presence of p25. This is in contrast to the previously reported translocation of BvIAA28 into cytoplasm upon co-expression with p25 (Gil et al., 2018). RT-qPCR results clearly demonstrated that the mRNA levels of *BvIAA2*, *BvIAA6* and *BvIAA28* did not show significant alterations at different stages of BNYVV infection as was tested at 28, 44 and 66 dpi. It can be speculated that p25 might exert a similar effect on BvIAA2 and BvIAA6 as the Rice dwarf virus (RDV) P2 protein on OsIAA10. RDV P2 manipulates the auxin signaling by targeting OsIAA10 in the nucleus and preventing its degradation by 26S proteasome (Jin et al., 2016; Qin et al., 2019). Contrary, the TMV replicase interacts with AtIAA26 and disrupts its nucleolar localization which affects the function of AtIAA26 as transcriptional repressor of auxin responsive genes (Padmanabhan et al., 2005, 2006, 2008). This mechanism appears to be similar to that exerted by p25 on the localization of BvIAA28, which is translocated into cytoplasm in the presence of p25 (Gil et al., 2018).

Attempts to identify the interaction domains in p25 and BvIAA2 and BvIAA6 yielded no results as small changes of the amino acid sequences led to a loss of interaction in either Y2H or BiFC, demonstrating the high specificity of the interaction. Even a single amino acid substitution in the NLS or NES signal of p25 disrupted the interaction. Since the expression of altered proteins used in the protein-interaction studies with p25 could be confirmed by immunoblotting we concluded that the interaction of p25 with BvIAA2 and BvIAA6 requires the full-length proteins. Viral proteins are multi-functional with an extensive networks of cellular interaction partners that has been developed during the co-evolution of viruses and their hosts (Callaway et al., 2001; Nagy, 2016; Valli et al., 2018). It has been observed in a previous study that sequence variation in the p25 protein affects its ability to self-interact and activate transcription in yeast one-hybrid system (Klein et al., 2007). Therefore, it can be speculated that sequence variation in p25 might affect its interaction with Aux/IAA proteins as these interactions seems to be very delicate and prone to disruption due to even slight alterations of the amino acid sequence. It was also not possible to identify interacting domains of BvIAA2 and BvIAA6 as was done for BvIAA28. BNYVV p25 appears to interact primarily with domains I and II of BvIAA28 (Gil et al., 2018). By contrast, the fact that similar approaches in identification potential interacting domains in BvIAA2 and BvIAA6 were not successful is probably due to some difference in the structure of BvIAA2 and BvIAA6 proteins compared to the BvIAA28 structure. Indeed, most of the Aux/IAA proteins contain extensive intrinsically disordered regions (IDRs), which are prone to conformational changes due to interaction with other proteins (Niemeyer et al., 2020). The presence of IDRs is a major factor promoting the interaction with multiple partners, thus, affecting interactions regulating stress responses, development, metabolic and signaling pathways (Covarrubias et al., 2020). On one hand, IDRs can provide structural flexibility for interaction and proper positioning of Aux/IAAs on e.g., Cullin RING-type E3 ubiquitin ligases TIR1 (Niemeyer et al., 2020). On the other hand, IDRs can be sensitive to changes of amino acid sequence when interacting with primarily ordered regions (Mishra et al., 2020) and the analysis predicts that p25 is an entirely ordered protein (data not shown). In order to make more precise statements in this regard and to determine possible interaction domains, further investigations, preferably with native proteins, might shed light on the nature of these interactions.

To conclude, in addition to BvIAA28, two sugar beet Aux/IAA proteins, namely, BvIAA2 and BvIAA6, were identified in this study to interact with p25, the BNYVV virulence factor. In contrast to BvIAA28, BvIAA2, and BvIAA6 do not appear to change their subcellular localization, they are not translocated into the cytoplasm by interaction with p25 and remain confined to the nucleus. Overall, the results show that p25 sequesters negative regulators of root development and thus likely promotes LR initiation and formation. The detailed mechanism of p25 action remains to be determined, hopefully with development of appropriate genetically tractable model systems as most genetic approaches in sugar beet are still extremely challenging and time consuming.

## Data Availability Statement

The original contributions presented in the study are included in the article/Supplementary Material, further inquiries can be directed to the corresponding author/s.

## Author Contributions

MV, SL, ES, and MM contributed to the conception and design of the study. MM performed and evaluated the practical work in the laboratory and wrote the first draft of the manuscript. MR performed the LC-MS/MS work and provided the data. All authors contributed to manuscript revision, read, and approved the submitted version.

## Conflict of Interest

The authors declare that the research was conducted in the absence of any commercial or financial relationships that could be construed as a potential conflict of interest.

## Publisher’s Note

All claims expressed in this article are solely those of the authors and do not necessarily represent those of their affiliated organizations, or those of the publisher, the editors and the reviewers. Any product that may be evaluated in this article, or claim that may be made by its manufacturer, is not guaranteed or endorsed by the publisher.
