# Supplementary material for: The Virulence Factor p25 of Beet Necrotic Yellow Vein Virus Interacts With Multiple Aux/IAA Proteins From Beta vulgaris: Implications for Rhizomania Development
Source: Front Microbiol. 2022 Jan 24;12:809690. doi: 10.3389/fmicb.2021.809690 (PMC8819154; doi:10.3389/fmicb.2021.809690)
Supplement: Supplementary file 1 [file Data_Sheet_1.docx]

**Supporting Information:**

1. **Figures**

**
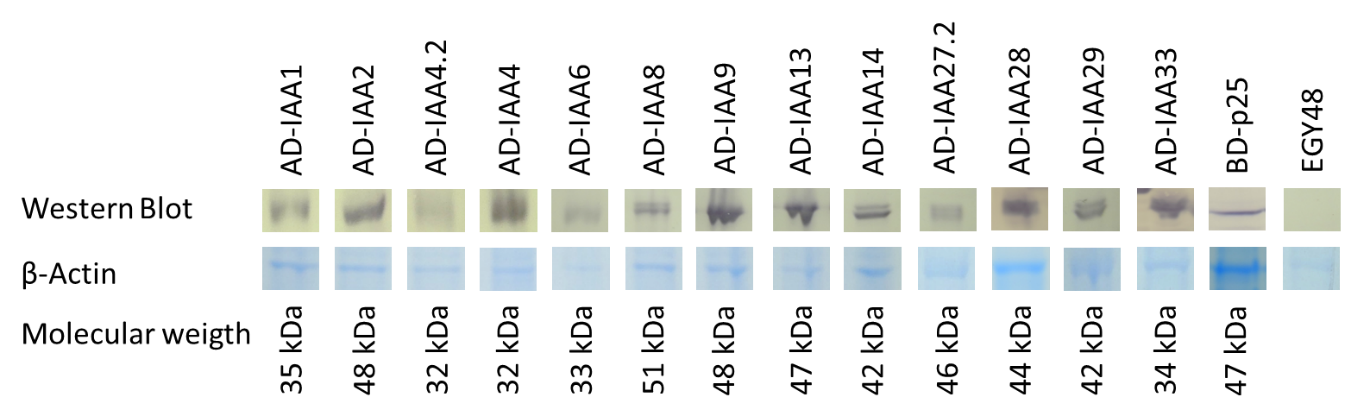
**

Figure S1. Expression of AD-Aux/IAA fusion proteins in yeast were detected using a HA tag and Expression of BD-p25 fusion proteins were detected using a LexA tag. Below the Western blot, β-actin (~ 43 kDa) is shown as loading control in Coomassie stained SDS gels. The molecular weight of each Aux/IAA protein is indicated below the loading controls. The yeast strain EGY48 without any plasmid served as negative control.


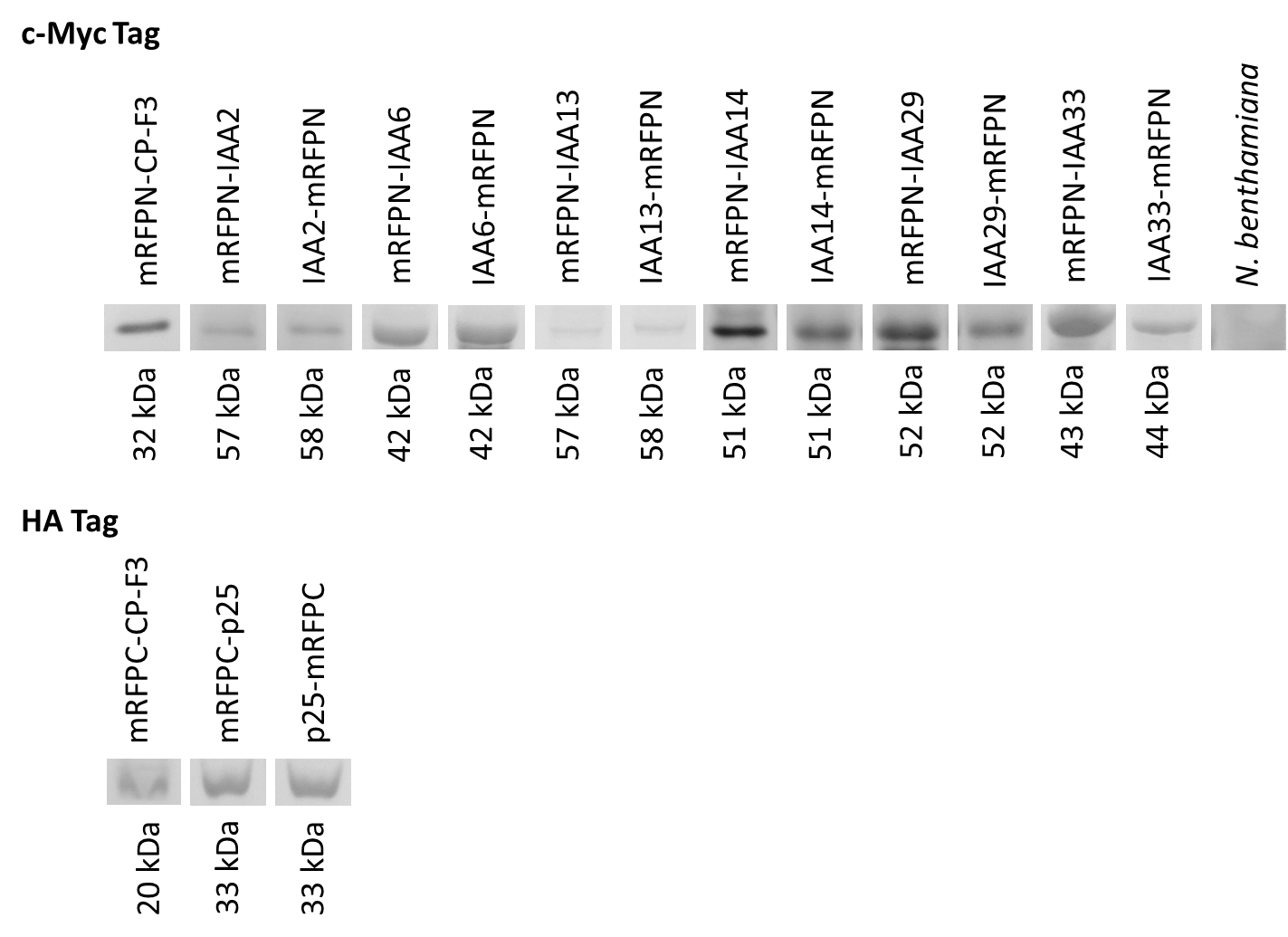


Figure S2. Detection of all fusion-proteins used in the BiFC assay by immunoblot. The name of each fusion protein, including the positive (+) and the negative (-) controls is given above each signal and the molecular weights of the proteins are given below. The upper part of the figure shows the immunoblot of C-Myc tagged fusion-proteins and the lower part shows HA tagged proteins. A protein sample from non-inoculated, healthy *N. benthamiana* leaves served as negative control to exclude unspecific binding of the antibodies. Images were taken at 4 dpi. Scale bars, 50 μm.


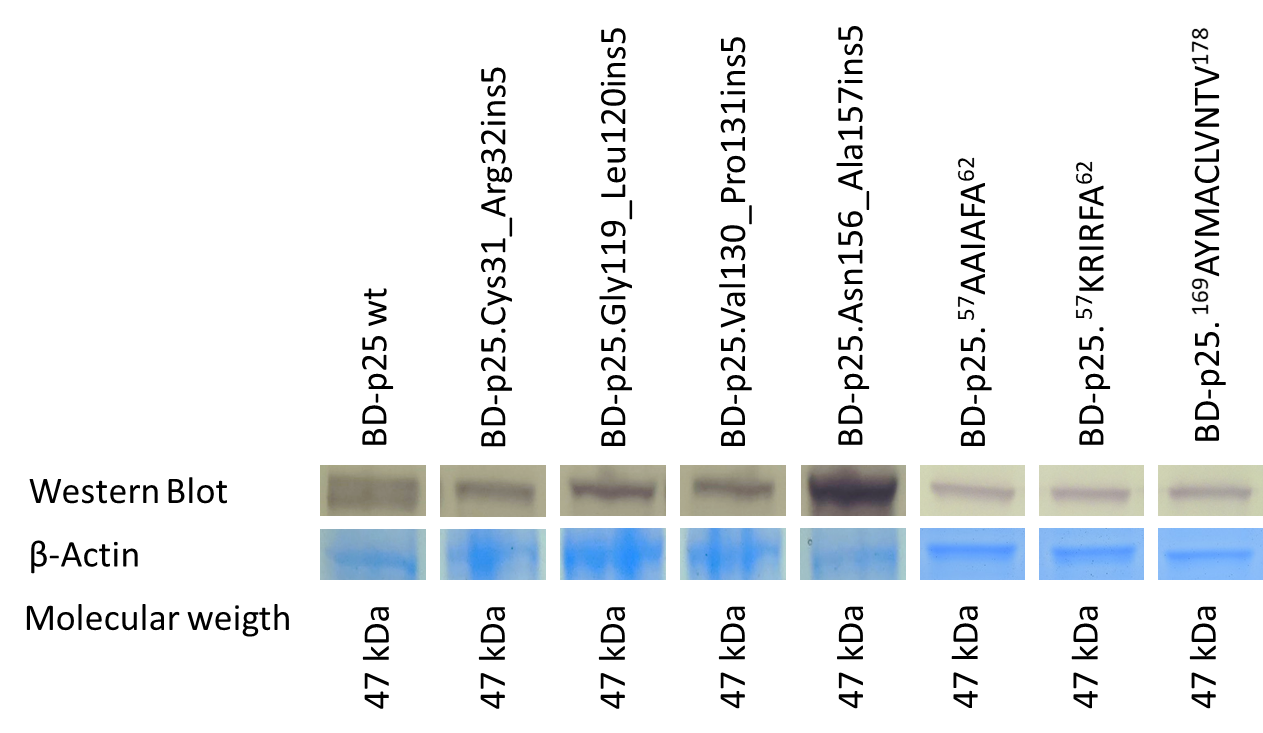


Figure S3. Detection of BD-p25 wt and different BD-p25 variants from the yeast experiments by immunoblot. The first four p25 fusion proteins variants after p25 wt were obtained from the Pentapeptide scanning mutagenesis and the last three fusion protein variants represent p25 varieties with mutated NLS and NES motifs. As in the other experiment fusion proteins with the BD were detected using a LexA. Below the Western blot, β-actin (~ 43 kDa) is shown as loading control in Coomassie stained SDS gels. The molecular weight of each p25 protein variant is indicated below the loading controls.


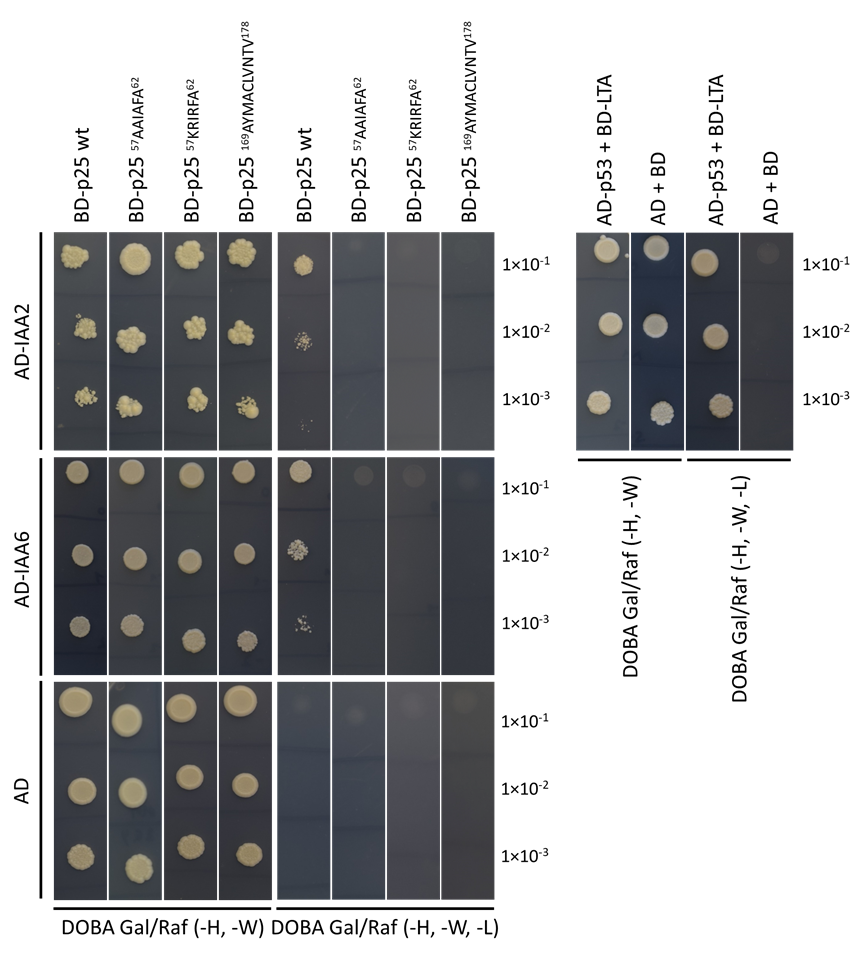


Figure S4. Results from a YTH experiment where IAA2 and IAA6 were tested for interaction with four BNYVV p25 varieties, p25 wt, p25 ^57^AAIAFA^62^, p25 ^57^KRIRFA^62^ and p25 ^169^AYMACLVNTV^178^. The positive control AD-p53 with BD-LTA and the negative control AD(-empty) with BD(-empty) were supplied by MoBiTech. BNYVV p25 was fused to the BD and the IAAs to the AD to test for interaction. Yeast transformants, containing both plasmids were selected on DOBA Glu (-H, -W), single colonies were resuspended in water and diluted 1×10^-1^- 1×10^-3^. 5 µl of each dilution was spotted on the control medium (DOBA Glu (-H, -W) and selection medium (DOBA Gal/Raf (-H, -W, -L)). AD without any fusion proteins and transformed with all BD-p25 varieties, served as control for autoactivation. AD - activating domain; BD - binding domain; DOBA – Dropout Base Agar.


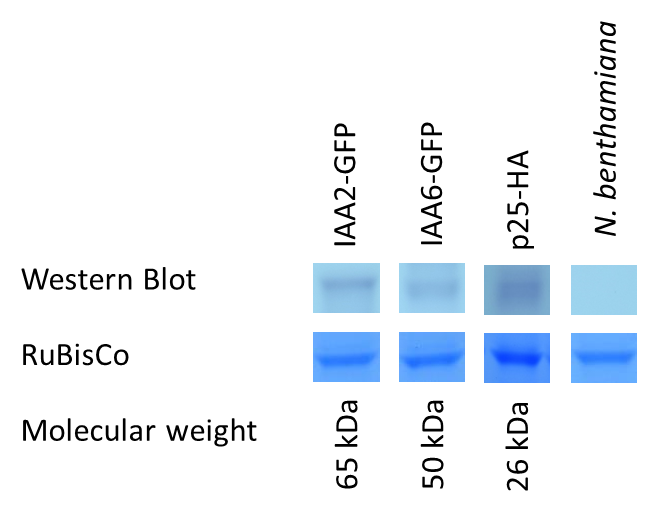


Figure S5. Detection of IAA2-GFP, IAA6-GFP and p25-HA by immunoblot. All fusion proteins were detected by HA antibodies. Tobacco Ribulose-1,5-bisphosphate carboxylase-oxygenase (RuBisCo) served as loading control) in Coomassie stained SDS gels (~ 55 kDa). The molecular weight of each Aux/IAA protein is indicated below the loading controls. A protein sample from non-inoculated, healthy *N. benthamiana* leaves served as negative control to exclude unspecific binding of the antibodies.


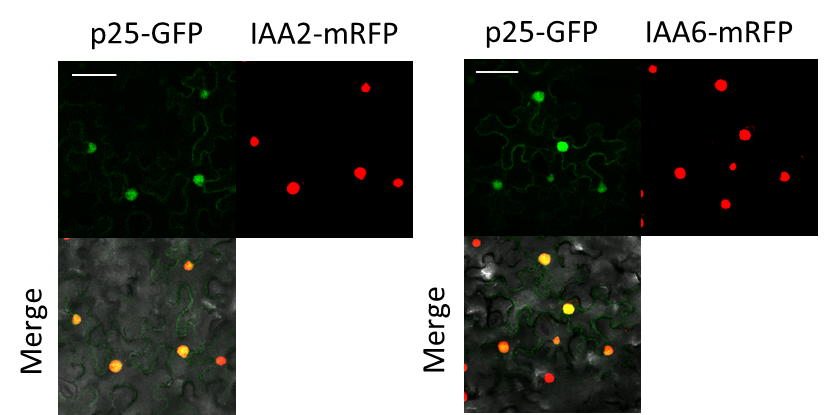


Figure S6. Subcellular localization of p25 fused to GFP (p25-GFP) co-expressed with the interacting Aux/IAAs fused to mRFP (IAA2-mRFP and IAA6-mRFP in *N. benthamiana* epidermal leaf cells. Images were taken at 4 dpi. Scale bars, 50 μm.

Figure S7. Mean absorbance values (A_405_) determined by double antibody sandwich ELISA in lateral roots of BNYVV inoculated and non-inoculated (mock) sugar beets used for qPCR quantification of *IAA2*, *IAA6* and *IAA28*. The plants were harvested after 28, 42 and 66 dpi. Vertical bars indicate SD (n=5).


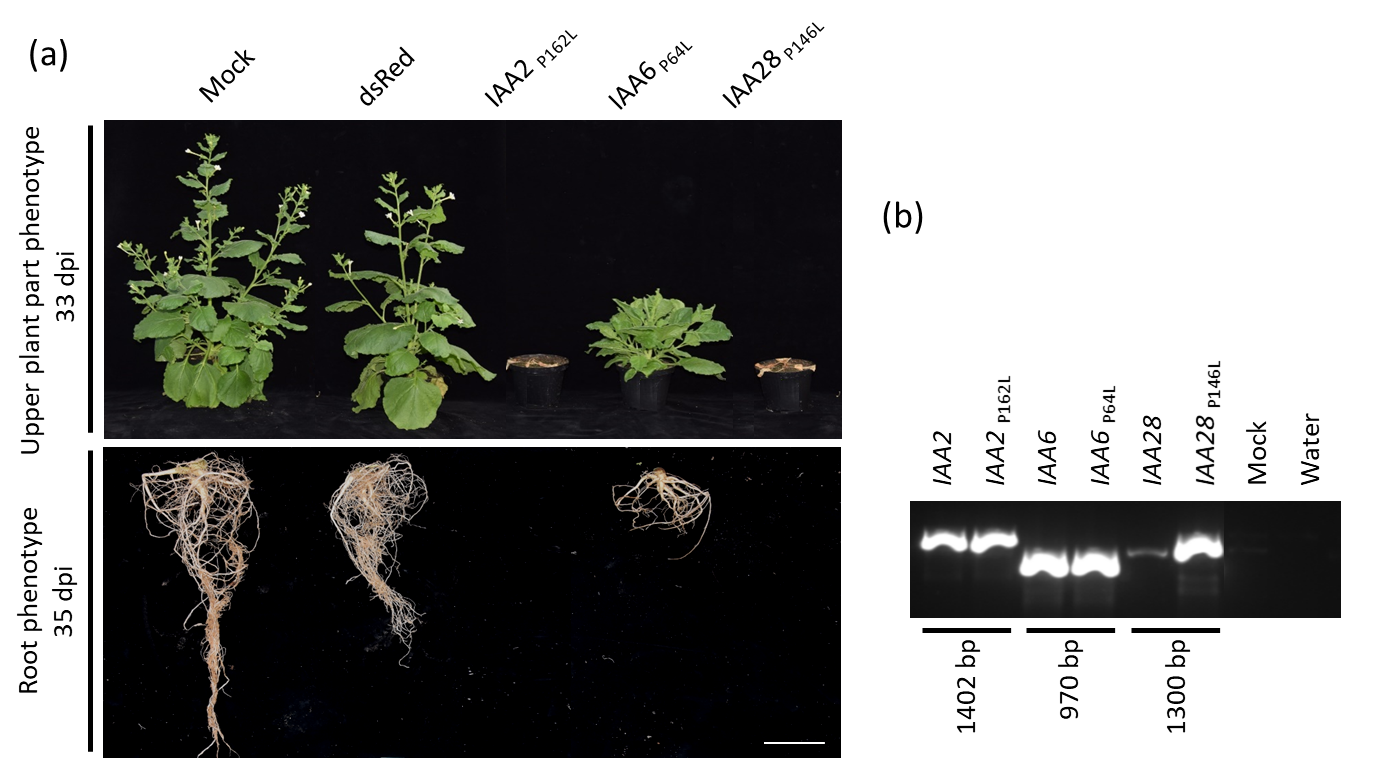


Figure S8. (A) Upper plant part and root phenotypes of *N. benthamiana*, non-inoculated (mock), infected with TRV expressing dsRed and infected the three TRV mutants overexpressing the degradation resistant Aux/IAA variants IAA2 _P162L_, IAA6 _P64L_ and IAA28 _P146L_. Pictures of the upper plant part were taken at 33 dpi, pictures of the root phenotype were taken at 35 dpi. Scale bar, 5 cm. (B) PCR amplification of all *Aux/IAA* genes in cDNA samples made from TRV systemically infected *N. benthamiana* leaf samples. The names of the genes are given above the signals and the size of the signals is given below the picture in base pairs (pb). cDNA from a non-inoculated *N. benthamiana* plant served as negative control (mock).

1. **Tables**

Table S1. List of all *IAA* genes from sugar beet

| Gene | KEGG Acc. No. | Oligonucleotide pair | Size (bp) |
| --- | --- | --- | --- |
| *IAA1* | 104904635 | #9 + #10 | 618 |
| *IAA2* | 104883127 | #11 + #12 | 996 |
| *IAA4* | 104890935 | #13 + #14 | 546 |
| *IAA4.2* | 104906976 | #15 + #16 | 534 |
| *IAA6* | 104904637 | #17 + #18 | 564 |
| *IAA8* | 104883520 | #19 + #20 | 1098 |
| *IAA9* | 104897812 | #21 + #22 | 1020 |
| *IAA13* | 104899391 | #23 + #24 | 978 |
| *IAA14* | 104894592 | #25 + #26 | 837 |
| *IAA27.2* | 104904711 | #29 + #30 | 945 |
| *IAA29* | 104901993 | #31 + #32 | 795 |
| *IAA32* | 104884870 | #33 + #34 | 594 |
| *IAA33* | 104902411 | #35 + #36 | 603 |

Table S2. List of all oligonucleotides used in this study. The Oligonucleotides are sorted according to their application (YTH, BiFC, qPCR, co-localization, and TRV-expression).

| Primer Name | Sequence (5' to 3') | |
| --- | --- | --- |
| **YTH** |  |  |
| #1 IAA28F | CTAGAATTCATGTTGAGTGCTGAGATTAGAGACACTTATAGCAC | |
| #2 IAA28R | CAGTCTCGAGTCAGCTTCTACTCTTGCATTTCTCGACAGC | |
| #3 p25 F | ATCGAATTCATGGGTGATATATTAGGCGC | |
| #4 p25 Atyp | TACCTCGAGCTAATCATCATCATCAACAC | |
| #6 BAIT seq. | CGTCAGCAGAGCTTCACC | |
| #7 PREY seq. | CTGAGTGGAGATGCCTCC | |
| #9 IAA1F | CTAGAATTCATGGAACAACAACAAGAAGT | |
| #10 IAA1R | TACCTCGAGGTTACTCAATGTTGGATGGTG | |
| #11 IAA2F | CTAGAATTCATGGGTGAAAGTAACCCAAA | |
| #12 IAA2R | TACCTCGAGTCACTTGGATGCACTCTC | |
| #13 IAA4F | CTAGAATTCATGGAGATGAACAAGAAAGAAA | |
| #14 IAA4R | TACCTCGAGTTAAGCCAAACAACCCAAG | |
| #15 IAA4.2F | CTAGAATTCATGTATAGGAAAGAAGATGATCAA | |
| #16 IAA4.2R | TACCTCGAGTTAGCATTTCTCCAAAGCAG | |
| #17 IAA6F | CTAGAATTCATGTCGAAAGCGGGTT | |
| #18 IAA6R | TACCTCGAGTCACCCATGGCATTGC | |
| #19 IAA8F | CTAGAATTCATGTCTGGTGTTAGAGAGGA | |
| #20 IAA8R | TACCTCGAGCTAGCTCCTGTTCCTGC | |
| #21 IAA9F | CTAGAATTCATGTCTCCCCCATTATTGG | |
| #22 IAA9R | TACCTCGAGCTAGTTCCGGCTCTTAGAT | |
| #23 IAA13F | CTAGAATTCATGGAAGCTGTAATGGGG | |
| #24 IAA13R | TACCTCGAGTTATATAGGCCGACTTCTTTG | |
| #25 IAA14F | CTAGAATTCATGGAAGTTGGGTTGATGAA | |
| #26 IAA14R | TACCTCGAGTTAGCTCCTGTTCTTGCA | |
| #27.2 IAA27F.2 | CATGTCAATGGGTTTTGAAGA |  |
| #28.2 IAA27R | TACCTCGAGTTAGCCATCGCTAACTCTTG |  |
| #29 IAA27.2F | CTAGAATTCATGTCTAGGCCATTAGAACA | |
| #30 IAA27.2R | TACCTCGAGTCAGGCTTCAGTCTTACAC | |
| #31 IAA29F | CTAGAATTCATGGAGCTTGAATTAGGTCT | |
| #32 IAA29R | TACCTCGAGTTAATCATCCCTTCTCCTTAGC | |
| #33 IAA32F | CTAGAATTCATGGAATCGAACATGGCA | |
| #34 IAA32R | TACCTCGAGGCAAAGAGGTTAAGGATTGT | |
| #35 IAA33F | CTAGAATTCATGTATAACAACATGAATAACAATAAGA | |
| #36 IAA33R | TACCTCGAGCTAGTGTTTTGTGCTCCTTT | |
| #39 pJG-Ins. seq. up | GACTGGCTGAAATCGAATGG | |
| #40 pJG-Ins. seq. Low | GCCGACAACCTTGATTG | |
| #280_pJG4-5Domain_fw | CTCGAGAAGCTTTGGACTTC | |
| #281_IAA2DI+II_rv | TCAACTCGGAATCGCACTC | |
| #282_IAA2_DIII+IV-fw | AAGCCTGTGAATGAAAAATCAG | |
| #284_IAA6DI+II_rv | TCACTCATTACCAATGCTCCTCC | |
| #285_IAA6_DIII+IV-fw | AAGGAATGTATTGAGGCATCAAAG | |
| #293_IAA6DII-IV_fw_new | GATATAGTTGGCGGCCAC | |
| #299_IAA6YTH_DI-III-fw | TGACTCGAGAAGCTTTGGAC | |
| **BiFC** |  |  |
| #44 pCB-mRFP-Nterm-fw | CGATCCTCTAGAGTCCGCAAAAATCACC | |
| #45 pCB-mRFP-Nterm-rv | CTCCACCAGATCCACCTCCGG | |
| #46 IAA28Rz2-N-fw | GATCTGGTGGAGGTGGATCCAGCAGCACGATTAATTTCGAAGAGACAGA | |
| #47 IAA28Rz2-N-rv | CTCTAGAGGATCGATCCTTAGCTTCTACTCTTGCATTTCTCGACAGC | |
| #48 mRFP-N-fw | CTACAAGACCGACATCAAGCTGGAC | |
| #49 mRFP-N-rv | CGAAACCCTATAAGAACCCTAATTCCCT | |
| #50 pCB-mRFP-Cterm-fw | GGAGGTGGATCTGGTGGAGGTAC | |
| #51 pCB-mRFP-Cterm-rv | GTGCTGCTTGTTATATCTCCTTCGAAGATCT | |
| #52 IAA28Rz2-C-fw | TCTTCGAAGGAGATATAACA ATG AGCAGCACGATTAATTTCGAAGAGAC | |
| #53 IAA28Rz2-C-rv | CCTCCACCAGATCCACCTCCGCTTCTACTCTTGCATTTCTCGACAG | |
| #54 mRFP-C-fw | TTCTCAACACAACATATACAAAACAAACGAATC | |
| #55 mRFP-C-rv | GGAGCCCTCCATGCGC | |
| #56 pCB-smRSGFP-Nterm-fw | TGAGTCCGCAAAAATCACCAGTCTCTC | |
| #57 pCB-smRSGFP-Nterm-rv | ACCTCCACCAGATCCACCTCCTTTGTAT | |
| #58 p25-N-fw | GAGGTGGATCTGGTGGAGGTATGGGTGATATATTAGGCGCAG | |
| #59 p25 ATyp-N-rv | TGGTGATTTTTGCGGACTCAACCATCATCATCAACACCGTC | |
| #60 smRSGFP-N-fw | CACAATCTGCCCTTTCGAAAGATCC | |
| #61 smRSGFP-N-rv | CCCTAATTCCCTTATCTGGGAACTAC | |
| #62 pCB-smRSGFP-Cterm-fw | GGAGGTGGATCTGGTGGAGG | |
| #63 pCB-smRSGFP-Cterm-rv | TGTTATATCTCCTTCGAAGATCTATCG | |
| #64 p25-C-fw | TCTTCGAAGGAGATATAACAATGGGTGATATATTAGGCGCAG | |
| #65 p25 A-Typ-C-rv | CCTCCACCAGATCCACCTCCACCATCATCATCAACACCGTC | |
| #66 smRSGFP-C-fw | GAAAATTTGTGCCCATTAACATCACC | |
| #67 smRSGFP-C-rv | CAATCCCACTATCCTTCGCAAGACC | |
| #69 Seq. pBIN19 fw | CAAAAGTTGATTTCTGAGGAGGATCTTGGT | |
| #70 Seq. pBIN19 rev | AAATTTTATTGATAGAAGTATT | |
| #71 pCB-mRFPN-Nterm-fw | GGATCCACCTCCACCAGATCCACC | |
| #72 pCB-mRFPN-Nterm-rv | TAAGGATCGATCCTCTAGAGTCCGCAAAAAT | |
| #73 pCB IAA28sus.N-fw | GATCTGGTGGAGGTGGATCCATGTTGAGTGCTGAGATTAGAGACACTTAT | |
| #74 pCB IAA28sus.-N-rv | CTCTAGAGGATCGATCCTTA TCAGCTTCTACTCTTGCATTTCTCGACAGC | |
| #75 pCB mRFPN-N-fw_seq | CTACAAGACCGACATCAAGCTGGAC | |
| #76 pCB mRFPN-N-rv_seq | ACATGAGCGAAACCCTATAAGAACCC | |
| #77 pCB-mRFPN-Cterm-fw | TAAGGATCGATCCTCTAGAGTCCGC | |
| #78 pCB-mRFPN-Cterm-rv | GGATCCACCTCCACCAGATCCA | |
| #79 pCB IAA28sus-C-fw | TCTTCGAAGGAGATATAACAATGTTGAGTGCTGAGATTAGAGACACTTATAGCAC | |
| #80 pCB IAA28sus-C-fv | CCTCCACCAGATCCACCTCCGCTTCTACTCTTGCATTTCTCGACAGC | |
| #81 pCB mRFPN-C-fw_seq | CTACAAGACCGACATCAAGCTGGAC | |
| #82 pCB mRFPN-C-rv_seq | ACATGAGCGAAACCCTATAAGAACCC | |
| #83 pCB IAA28 Rz2 (-10AS)N-fw | GATCTGGTGGAGGTGGATCCAGCAGCACGATTAATTTCGAAGAGACAGA | |
| #84IAA28 Rz2 (-10AS)-C-fw | TCTTCGAAGGAGATATAACAAGCAGCACGATTAATTTCGAAGAGACAGA | |
| #85 pCB-mRFPC-Nterm-fw | TAAGGATCGATCCTCTAGAGTCCGC | |
| #86 pCB-mRFPC-Nterm-rv | GGATCCACCTCCACCAGATCC | |
| #87 pCB p25 BTyp -N-fw | GATCTGGTGGAGGTGGATCCATGGGTGATATATTAGGCGCAGTTTAT | |
| #88pCB p25 BTyp -N-rv | CTCTAGAGGATCGATCCTTACTAATCATCATCATCAACACCGTCAGG | |
| #89 pCB p25 BTyp-C-fw | TCTTCGAAGGAGATATAACAATGGGTGATATATTAGGCGCAGTTT | |
| #90 pCB p25 BTyp-C-rv | CCTCCACCAGATCCACCTCCATCATCATCATCAACACCGTCAGG | |
| #91 pCB mRFPC-C-fw_seq | ACATGAGCGAAACCCTATAAGAACCC | |
| #110 IAA28sus. F BiFC | TGAGGATCCATGTTGAGTGCTGAGATTAGAGACAC | |
| #111 IAA28sus.+Rz2(10AS) R (stopp) BiFC | CTAGTCGACTCAGCTTCTACTCTTGCATTTCTC | |
| #112 IAA28sus.+Rz2(10AS) R BiFC | CTAGTCGACGCTTCTACTCTTGCATTTCTCGAC | |
| #114 pCB mRFPN GOI Seq. | CTCCACCGAGCGGATGTAC | |
| #115 pCB GOI mRFPN Seq. | CTCAAGCAATCAAGCATTCTAC | |
| #134 IAA2 F BiFC | TGAGGATCCATGGGTGAAAGTAACCCAAA | |
| #135 IAA2 R Stopp BiFC | CTAGTCGACCTTGGATGCACTCTCCAC | |
| #136 IAA2 R BiFC | CTAGTCGACTCACTTGGATGCACTCTC | |
| #137 IAA6 F BiFC | TGAGGATCCATGTCGAAAGCGGGT | |
| #138 IAA6 R Stopp BiFC | CTAGTCGACCCCATGGCATTGCTTC | |
| #139 IAA6 R BiFC | CTAGTCGACTCACCCATGGCATTGC | |
| #140 IAA13 F BiFC | TGAGGATCCATGGAAGCTGTAATGGGG | |
| #141 IAA13 R Stopp BiFC | CTAGTCGACTATAGGCCGACTTCTTTGC | |
| #142 IAA13 R BiFC | CTAGTCGACTTATATAGGCCGACTTCTTTGC | |
| #143 IAA14 F BiFC | TGAGGATCCATGGAAGTTGGGTTGATGA | |
| #144 IAA14 R Stopp BiFC | CTAGTCGACTTAGCTCCTGTTCTTGCAC | |
| #145 IAA14 R BiFC | CTAGTCGACGCTCCTGTTCTTGCAC | |
| #146 IAA29 F BiFC | TGAGGATCCATGGAGCTTGAATTAGGTCTTTC | |
| #147 IAA29 R Stopp BiFC | CTAGTCGACTTAATCATCCCTTCTCCTTAGC | |
| #148 IAA29 R BiFC | CTAGTCGACATCATCCCTTCTCCTTAGC | |
| #149 IAA33 F BiFC | TGAGGATCCATGTATAACAACATGAATAACAATAAGAC | |
| #150 IAA33 R Stopp BiFC | CTAGTCGACCTAGTGTTTTGTGCTCCTTTG | |
| #151 IAA33 R BiFC | CTAGTCGACGTGTTTTGTGCTCCTTTGC | |
| #212mRFPN-GOIfw | GTCGACTAAGGATCGATCCT | |
| #213mRFPN-GOIrv | GGATCCACCTCCACCAG | |
| #214GOI-mRFPNfw | GTCGACGGAGGTGGATCTGG | |
| #215GOI-mRFPNrv | GGATCCCATTGTTATATCTCCTTCG | |
| #286_pBiFC _Domain_fw | GTCGACTAAGGATCGATCCTC | |
| #287_ pBiFC_Domain_rv | GGATCCACCTCCACCA | |
| #294_pBiFC_IAA6DI,III+IV_rv | GCCAACTATATCGGAAAACACC | |
| #295_pBiFC_IAA6DI+II,IV_fw | GAGGCATTGAAAGATGCG | |
| #296_pBiFC_IAA6DI+II,IV_rv | CTTTGATGCCTCAATACATTCC | |
| #297_pBiFC_IAA6DI-III _rv | CGCATCTTTCAATGCCTC | |
| #298_ pBiFC_IAA6DI-III_fw | TGAGTCGACTAAGGATCGATC | |
| #316_mRFPC-p25-rev | GACTCTAGAGGATCGATCCTTAACCATCATCATCAACACCGTC | |
| #298_ pBiFC_IAA6DI-III_fw | TGAGTCGACTAAGGATCGATC | |
| #316_mRFPC-p25-rev | GACTCTAGAGGATCGATCCTTAACCATCATCATCAACACCGTC | |
| **qPCR** |  |  |
| #118 IAA2 qPCR fw. | CACAGCCCTGTTGCACTAGA | |
| #119 IAA2 qPCR rev. | AATTGGAGGCCAACCCACAA | |
| #120 IAA6 qPCR fw. | GCATGGATGGTGTGCCTTTC | |
| #121 IAA6 qPCR rev. | CCGCATCTTTCAATGCCTCG | |
| #348_GAPDHqPCR | CACCACCGATTACATGACATACA | |
| #349_GAPDHqPCR7R | GGATCTCCTCTGGGTTCCTG | |
| #350_EF1AlphaqPCR7F | GCTTTTGAGGATCTCTGGCG | |
| #351_EF1Alpha qPCR7R | AAGCCTTAGAGTCAGCTGCT | |
| **co-localization** |  |  |
| #218pCBmRFP-GOIfw. | ATGGTCGACTAAGGATCGATCCTCTAGAGTC | |
| #219pCBmRFP-GOIrv. | GTAGGATCCACCTCCACCAGATCCAC | |
| #221pCB GOI-mRFP fw. | ATGGTCGACGGAGGTGGATCTGGTGG | |
| #222pCB GOI-mRFP rv. | GTAGGATCCTGTTATATCTCCTTCGAAGATCTATC | |
| #304_p25-pCB_rv | TGATTTTTGCGGACTCTAGATTAACCATCATCATCAACACCG | |
| #305_GSlink-p25_fw | GAGGTGGATCTGGTGGAGGTATGGGTGATATATTAGGCGCAG | |
| #306_p25-GS-link_rv | CCTCCACCAGATCCACCTCCACCATCATCATCAACACCG | |
| #307_IAA2-GS-link_rv | CCTCCACCAGATCCACCTCCCTTGGATGCACTCTCCACCA | |
| #308_GOI-pCB-fw | TGAGTCCGCAAAAATCACC | |
| #309_IAA2_pCB_rv | TGGTGATTTTTGCGGACTCACTTGGATGCACTCTCCACC | |
| #310_IAA6_pCB_rv | TGGTGATTTTTGCGGACTCACCCATGGCATTGCTTC | |
| #314_HA-35s | CTGACTATGCGTGATTCTCCAGAATAATGTGTGAG | |
| #315_GS-HA | GAACATCGTATGGGTAACCTCCACCAGATCCAC | |
| #317_pCBfwds | GACGGCCACTACGACGC | |
| #318_HArev | AGCGTAATCTGGAACATCGTATGG | |
| #319_dsRed-pCB_fw | ACGATGTTCCAGATTACGCTATGGTGCGCTCCTCCAAG | |
| #320_HA-dsRed_rv | TCGGCGTCGTAGTGGCCGTCTTACAGGAACAGGTGGTGGCG | |
| #321_dsRedGS_rv | CCTCCACCAGATCCACCTCCCAGGAACAGGTGGTGGC | |
| #322_GFP-SV40_fw | AAGAAAGGTTTGATTCTCCAGAATAATGTG | |
| #323- GFP-SV40-rv | TTCTTTTTTGGGTACAGCTCGTCCATG | |
| **TRV** |  |  |
| AUX2_fw | CTTACCCGAGTTAACGAGCCATGGGTGAAAGTAACCCAAAATTG | |
| AUX2_rv | CTCGGTACCGAGCTCGAATTCTACTTGGATGCACTCTCCACCA | |
| AUX6_fw | CTTACCCGAGTTAACGAGCCATGTCGAAAGCGGGTTTCGAAC | |
| AUX6_rv | CTCGGTACCGAGCTCGAATTCTACCCATGGCATTGCTTCTTTGACTG | |
| AUX28_fw | CTTACCCGAGTTAACGAGCCATGTTGAGTGCTGAGATTAGA | |
| AUX28_rv | CTCGGTACCGAGCTCGAATTCTAGCTTCTACTCTTGCATTTCTC | |
| AUX2_L162P-fw | TTCCAATTCGATCGTTCCGAAAG | |
| AUX2_L162P-rv | GCCAACCCACAACTGGAGTAG | |
| AUX6_L64P-fw | TAGGAGGAGGAGCATTGGTAATG | |
| AUX6_L64P-rv | TATGAGCACACTGGAAGCCAC | |
| AUX28_L146P-fw | CCAGTTCGAGCATTCAGGAAAC | |
| AUX28_L146P-rv | TAGCCAACCTACGACTTGTGC | |
